# Supplementary figures and images for: Synthesis of encapsulated ZnO nanowires provide low impedance alternatives for microelectrodes
Source: PLoS One. 2022 Jun 16;17(6):e0270164. doi: 10.1371/journal.pone.0270164 (PMC9202946; doi:10.1371/journal.pone.0270164)

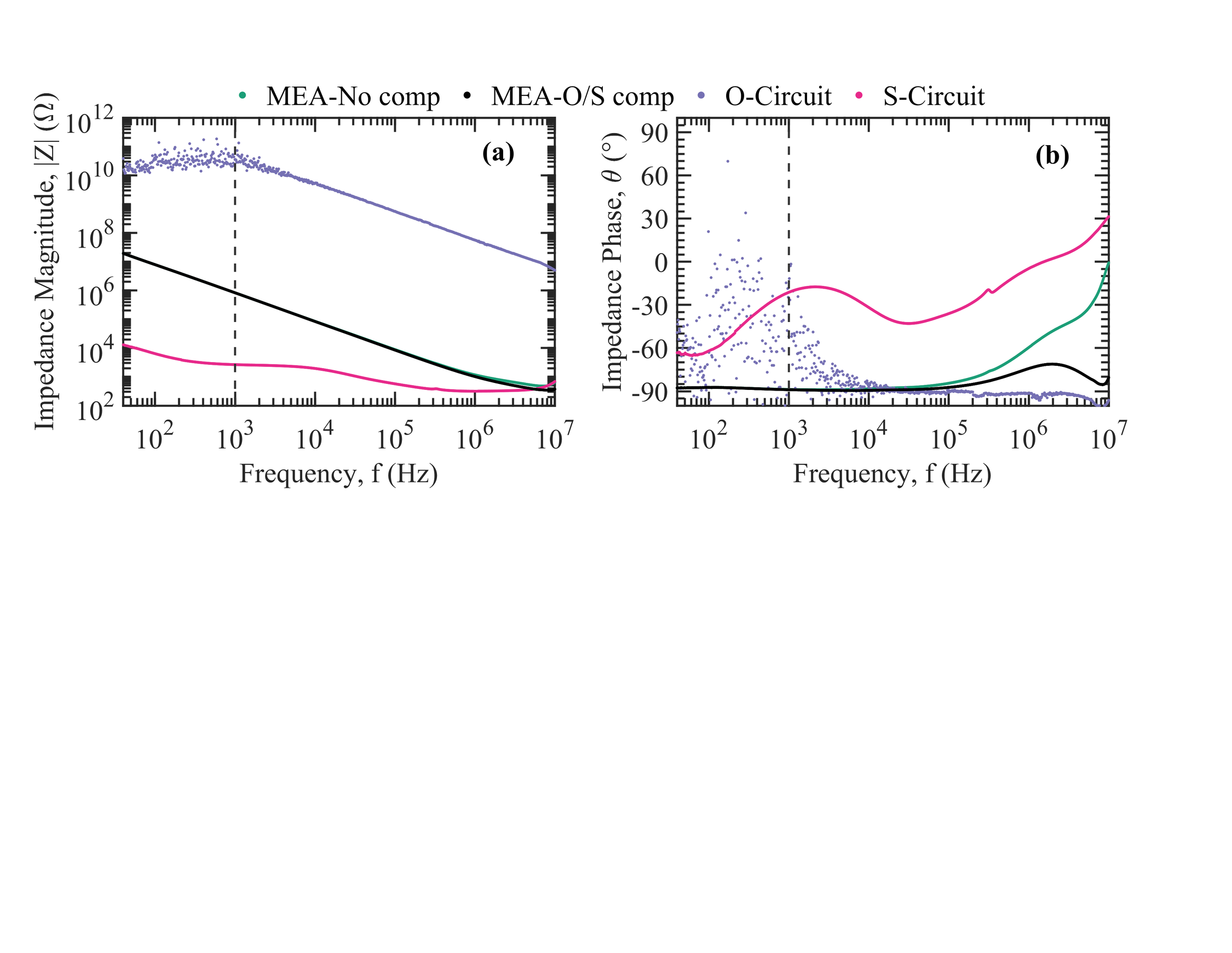

Supplement: S1 Fig — Electrochemical impedance spectroscopy bode plots as (a) impedance magnitude vs frequency and (b) impedance phase vs frequency of planar microelectrodes. The average impedance of planar microelectrodes before and after the open/short compensation are indicated by ‘MEA-No comp’ and ‘MEA-O/S comp’, respectively. The measured impedance of the open-circuit ‘O-Circuit’ and the short-circuit ‘S-Circuit’ that were used for the open/short compensation are also plotted in (a) and (b). Dashed lines indicate the frequency of 1 kHz. (TIF) [file pone.0270164.s001.tif]

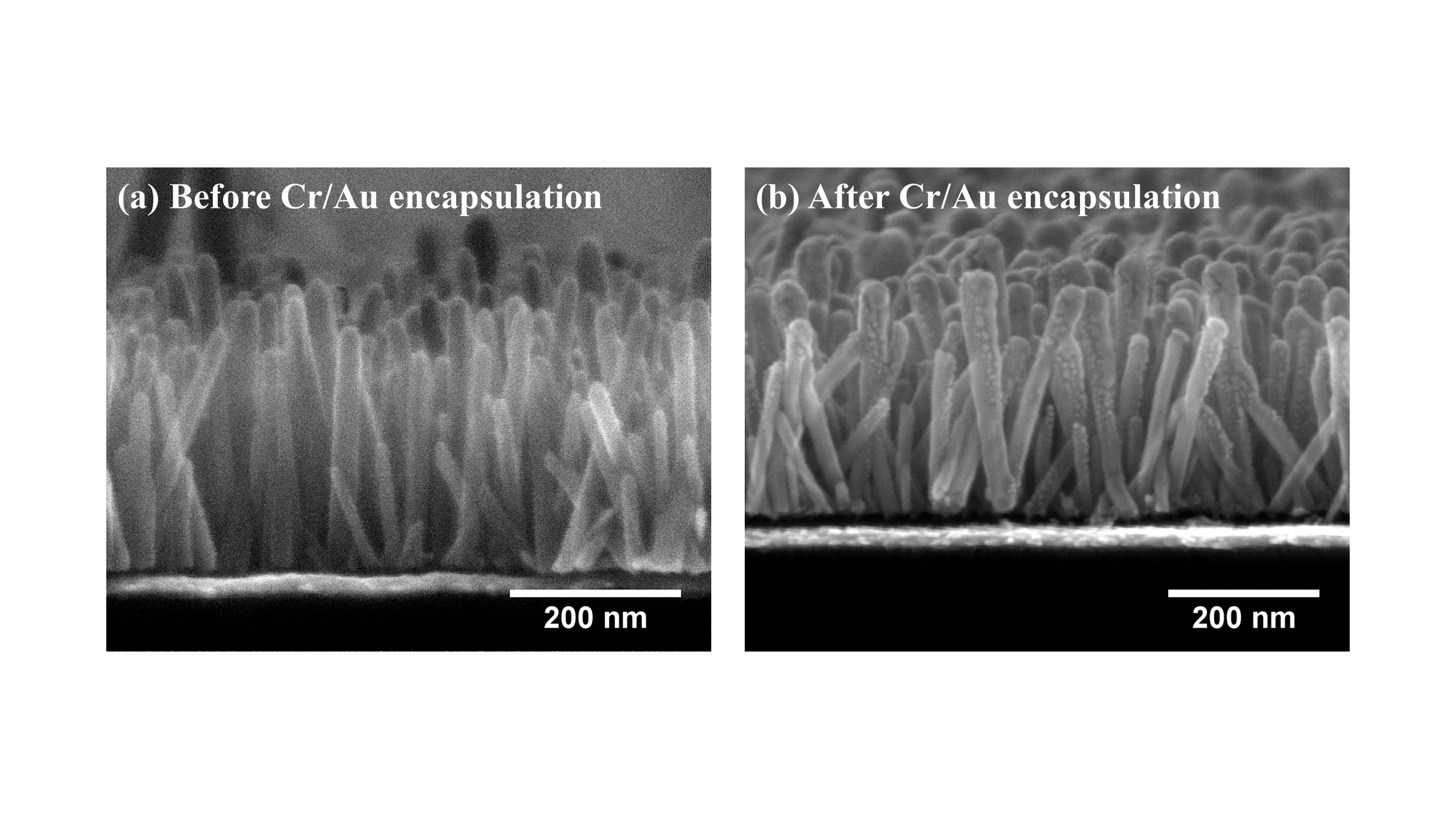

Supplement: S2 Fig — Cross-sectional scanning electron microscopy (SEM) of ZnO NWs (a) before and (b) after encapsulation with Cr/Au (2/20 nm) through the thermal evaporation that was applied perpendicular to the substrate plane. While the tops of the NWs are fully encapsulated, the lower regions are partially covered by the Cr/Au layer. (TIF) [file pone.0270164.s002.tif]

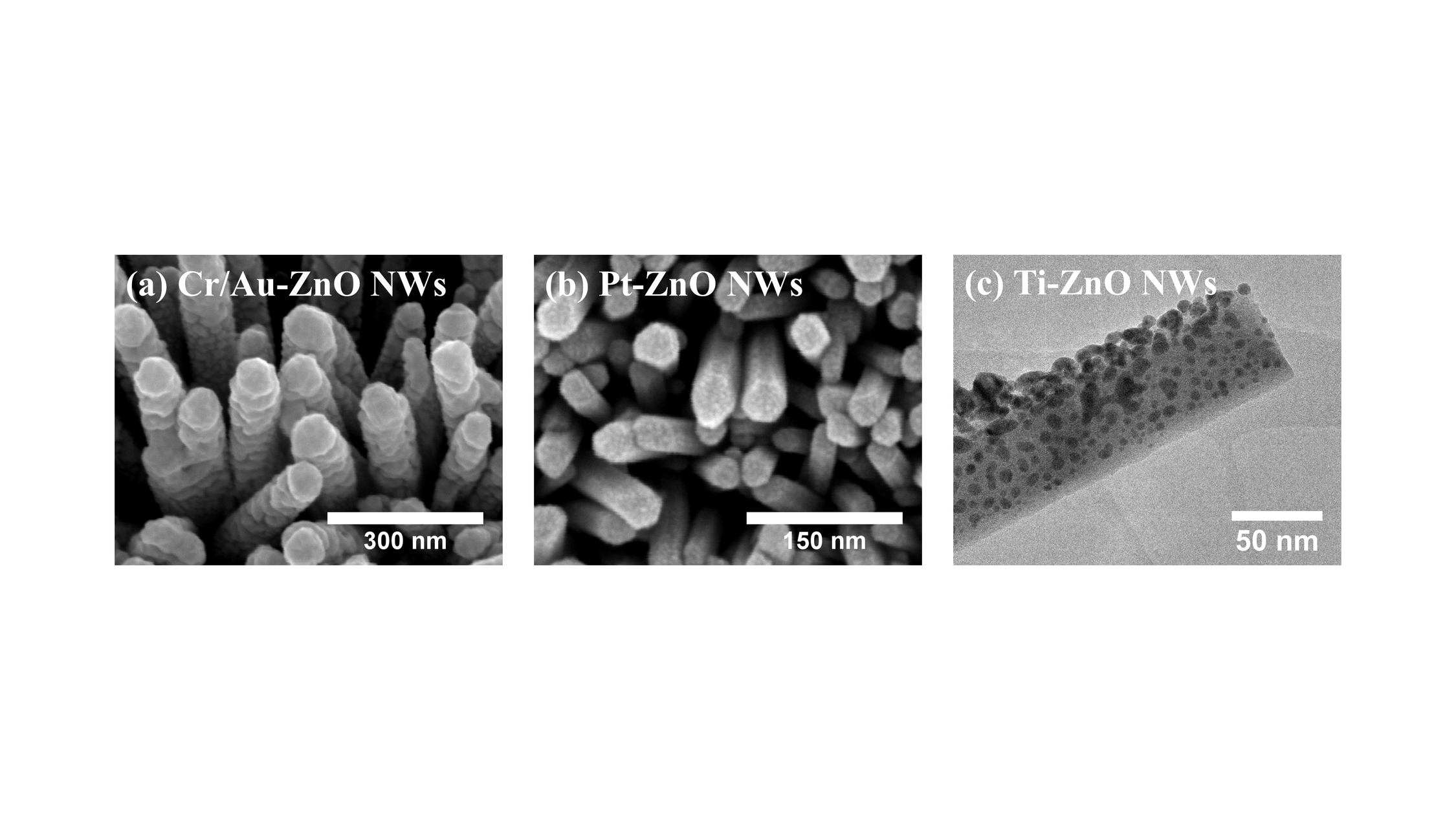

Supplement: S3 Fig — Scanning electron microscopy (SEM) of ZnO NWs encapsulated with (a) Cr/Au (2/20 nm) and (b) Pt (10 nm). (c) Transmission electron microscopy (TEM) of ZnO NWs encapsulated with Ti (10 nm). (TIF) [file pone.0270164.s003.tif]

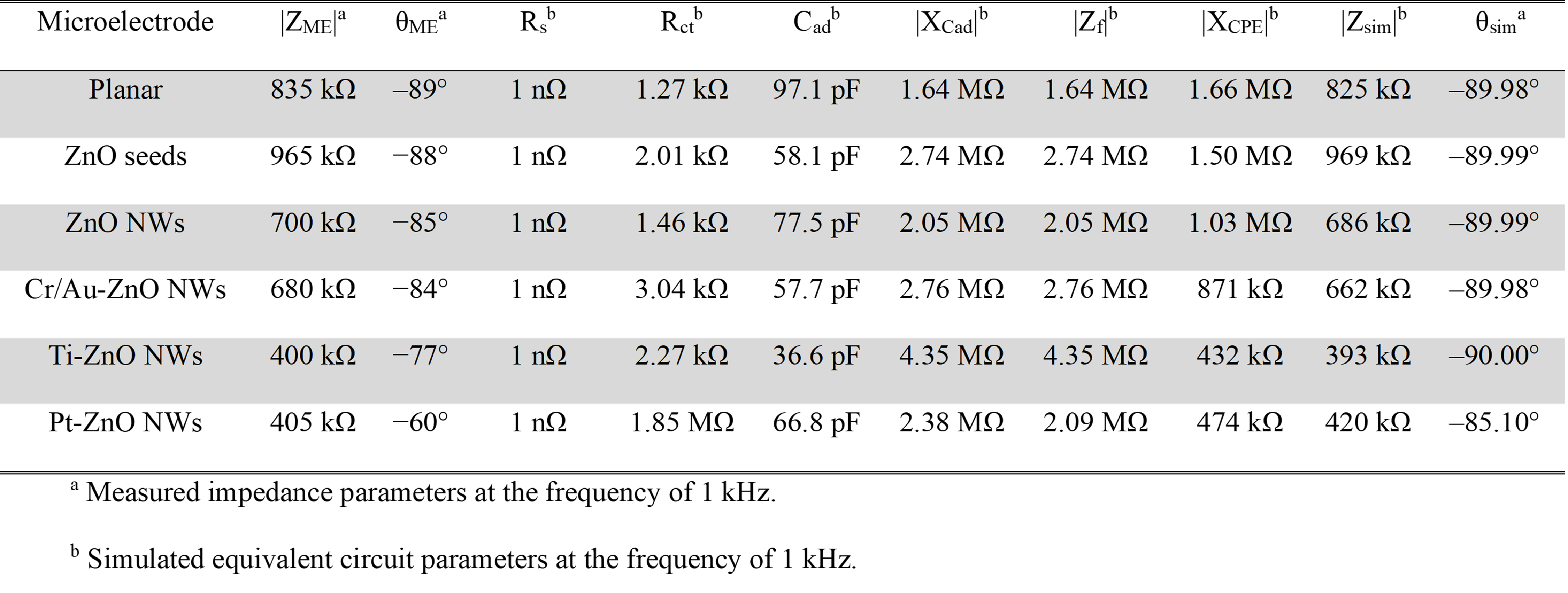

Supplement: S2 Table — Parameters are measured by fitting the measured impedance (after the open/short circuit compensation) to the modified Randles equivalent circuit. (TIF) [file pone.0270164.s005.tif]
