# Supplementary material for: Synthesis of encapsulated ZnO nanowires provide low impedance alternatives for microelectrodes
Source: PLoS One. 2022 Jun 16;17(6):e0270164. doi: 10.1371/journal.pone.0270164 (PMC9202946; doi:10.1371/journal.pone.0270164)
Supplement: S1 Table — (PDF) [file pone.0270164.s004.pdf]

Microelectrode	Nanomaterial	Nanomaterial size	Impedance (kΩ) at 1 kHz	Impedance for unit area ($Ω·cm^2$)	[Ref]
Ti/Au, 50 µm (dia)	Au nanowires	Ø: 10–1000 nm, L: 5 µm	500 kΩ	9.82 $Ω·cm^2$	[1]
Cr/Ni, 30 µm (dia)	Au nanopillars	Ø: 4–200 nm, L: 4–22.5 µm	13–360 kΩ	0.092–2.54 $Ω·cm^2$	[2]
Pt, 120 µm (dia)	Pt nanowires	600× increased surface area	2 kΩ	0.226 $Ω·cm^2$	[3]
Ti/Pt, 5–10 µm (sq)	Pt nanopillars	Ø: 150 nm, L: 1.5 µm	6 MΩ	1.5–6 $Ω·cm^2$	[4]
Au, 50 µm (dia)	Si/SiO2 nanowires with Ti/Au tips	Ø: 150 nm, L: 3 µm	N/A	N/A	[5]
P-doped poly-silicon, 30 µm (sq)	Vertically aligned MW-CNTs	Ø: 30–50 nm, L: 35 µm	3–4 kΩ	0.027–0.036 $Ω·cm^2$	[6]
TiN, 80 µm (dia)	MW-CNT islands	Ø: tens of nm	1.1–10 kΩ	0.0553–0.503 $Ω·cm^2$	[7]
Pt, 40 µm (diam)	SW-CNTs	Ø: 10–25 nm, L: 1–1.5 µm	20 kΩ	0.251 $Ω·cm^2$	[8,9]
Au,50 µm (dia)	MW-CNTs functionlised with carboxylic acid	Ø: 9.5 nm, L: 1.5 µm	19 kΩ	0.373 $Ω·cm^2$	[10]
Carbon film, 30 µm (dia)	Carbon nanofiber (CNF)	Ø: 23.6 µm, L: 20.7 µm	25 kΩ	0.177 $Ω·cm^2$	[11]
Au, 800 µm (sq)	N/A	N/A	17.9 kΩ	115 $Ω.cm^2$	[12,13]
Au, 800 µm (sq)	ZnO-NWs	Ø: 55 nm, L: 3.5 µm	1.45 kΩ	9.28 $Ω.cm^2$	[12,13]
Au, 800 µm (sq)	ZnO-NWs/ Cr/Au	Ø: 55 nm, L: 3.5 µm	636 Ω	4.07 $Ω.cm^2$	[12,13]

Au, 800 µm (sq)	ZnO-NWs/ Cr/Au/PEDOT	Ø: 55 nm, L: 3.5 µm	520 Ω	3.33 $\Omega.cm^2$	[12,13]
Cr/Au, 50 µm (dia)	N/A	N/A	835 ± 40 kΩ	16.4 ± 0.79 $\Omega.cm^2$	This Work
Cr/Au, 50 µm (dia)	ZnO-NWs	Ø: 75 ± 23 nm, L: 1.88 ± 0.178 µm	700 ± 40 kΩ	13.7 ± 0.79 $\Omega.cm^2$	This Work
Cr/Au, 50 µm (dia)	ZnO-NWs/ Cr/Au	Ø: 75 ± 23 nm, L: 1.88 ± 0.178 µm	680 ± 10 kΩ	13.4 ± 0.2 $\Omega.cm^2$	This Work
Cr/Au, 50 µm (dia)	ZnO-NWs/ Ti or Pt	Ø: 75 ± 23 nm, L: 1.88 ± 0.178 µm	400 ± 25 kΩ	7.85 ± 0.49 $\Omega.cm^2$	This Work

(dia): diameter of the circular electrode, (sq): width of the square electrode, (N/A): not available/stated

[1] Yoon H, Deshpande DC, Ramachandran V, Varadan VK. Aligned nanowire growth using lithography-assisted bonding of a polycarbonate template for neural probe electrodes. Nanotechnology 2007;19:25304.

[2] Nick C, Quednau S, Sarwar R, Schlaak HF, Thielemann C. High aspect ratio gold nanopillars on microelectrodes for neural interfaces. Microsyst Technol 2014;20:1849–57.

[3] Jin Y-H, Daubinger P, Fiebich BL, Stieglitz T. A novel platinum nanowire-coated neural electrode and its electrochemical and biological characterization. Micro Electro Mech. Syst. (MEMS), 2011 IEEE 24th Int. Conf., IEEE; 2011, p. 1003–6.

[4] Xie C, Lin Z, Hanson L, Cui Y, Cui B. Intracellular recording of action potentials by nanopillar electroporation. Nat Nanotechnol 2012;7:185–90.

[5] Robinson JT, Jorgolli M, Shalek AK, Yoon M-H, Gertner RS, Park H. Vertical nanowire electrode arrays as a scalable platform for intracellular interfacing to neuronal circuits. Nat Nanotechnol 2012;7:180–4.

[6] Wang K, Fishman HA, Dai H, Harris JS. Neural stimulation with a carbon nanotube microelectrode array. Nano Lett 2006;6:2043–8.

[7] Gabay T, Ben-David M, Kalifa I, Sorkin R, Ze'ev RA, Ben-Jacob E, et al. Electro-chemical and biological properties of carbon nanotube based multi-electrode arrays. Nanotechnology 2007;18:35201.

[8] Gabriel G, Gómez-Martínez R, Villa R. Single-walled carbon nanotubes deposited on surface electrodes to improve interface impedance. Physiol Meas 2008;29:S203.

[9] Gabriel G, Gómez R, Bongard M, Benito N, Fernández E, Villa R. Easily made single-walled carbon nanotube surface microelectrodes for neuronal applications. Biosens Bioelectron 2009;24:1942–8.

[10] Nick C, Thielemann C. Are carbon nanotube microelectrodes manufactured from dispersion stable enough for neural interfaces? Bionanoscience 2014;4:216–25.

[11] Fang S-P, Jao PF, Franca E, DeMarse TB, Wheeler BC, Yoon Y-K. A carbon nanofiber (CNF) based 3-D microelectrode array for in-vitro neural proliferation and signal

recording. 2016 IEEE 29th Int. Conf. Micro Electro Mech. Syst., IEEE; 2016, p. 423–6.

[12] Ryu M, Yang JH, Ahn Y, Sim M, Lee KH, Kim K, et al. Enhancement of interface characteristics of neural probe based on graphene, ZnO nanowires, and conducting polymer PEDOT. ACS Appl Mater Interfaces 2017;9:10577–86. https://doi.org/10.1021/acsami.7b02975.

[13] Ryu M, Lee KH, Sim M, Kim S, Jun B-O, Jang JE, et al. Nanowire based flexible electrode array with pedot film for neural recordings. 2015 IEEE 15th Int. Conf. Nanotechnol., IEEE; 2015, p. 868–71. https://doi.org/10.1109/NANO.2015.7388751.
