# Supplementary material for: Synthesis of encapsulated ZnO nanowires provide low impedance alternatives for microelectrodes
Source: PLoS One. 2022 Jun 16;17(6):e0270164. doi: 10.1371/journal.pone.0270164 (PMC9202946; doi:10.1371/journal.pone.0270164)
Supplement: S1 File — (PDF) [file pone.0270164.s006.pdf]

The total simulated impedance of the equivalent circuit model can be calculated as below

$$Z_{sim} = R_s + (Z_f \parallel Z_{nf}) \quad (1),$$

where $Z_f$ is the Faradaic impedance as the series combination of the charge transfer resistance ($R_{ct}$) and the reactance of the adsorption capacitance ($X_{Cad}$)

$$Z_f = R_{ct} + jX_{Cad} \quad (2),$$

$$X_{Cad} = \frac{1}{2\pi f C_{ad}} \quad (3),$$

and $Z_{nf}$ is the non-Faradaic impedance

$$Z_{nf} = jX_{CPE} \quad (4),$$

with $X_{CPE}$ as the reactance of the constant phase element (CPE)

$$X_{CPE} = \frac{1}{Q\,(2\pi f)^n} \quad (5),$$

where n is the constant exponent, $0 \leq n \leq 1$, with 1 representing an ideal capacitor and 0 representing an ideal resistor. The Q is the constant coefficient with the unit of $F \cdot cm^{-2} \cdot s^{n-1}$ that represents the double-layer capacitance at the angular frequency of 1 rad/s.

The total simulated impedance (equation 1) can subsequently be expressed as

$$Z_{sim} = R_s + \frac{Z_f\, Z_{nf}}{Z_f + Z_{nf}} \quad (6),$$

$$= R_s + \frac{(R_{ct} + jX_{Cad})\, jX_{CPE}}{R_{ct} + j\,(X_{Cad} + X_{CPE})} \quad (7),$$

$$= R_s + \frac{R_{ct}\, X_{CPE}^2}{R_{ct}^2 + (X_{Cad} + X_{CPE})^2} + j\left(\frac{R_{ct}^2\, X_{CPE} + X_{Cad}^2\, X_{CPE} + X_{Cad}\, X_{CPE}^2}{R_{ct}^2 + (X_{Cad} + X_{CPE})^2}\right) \quad (8)$$

The real and imaginary part of the simulated impedance can be separated as $Z_{real}$ and $Z_{img}$, respectively, to calculate the simulated impedance magnitude ($|Z_{sim}|$) and phase ($\theta_{sim}$) using the equations below.

$$Z_{real} = R_s + \frac{R_{ct}\, X_{CPE}^{\;2}}{R_{ct}^{\;2} + (X_{Cad} + X_{CPE})^2} \qquad (9),$$

$$Z_{img} = \frac{R_{ct}^{\;2}\, X_{CPE} + X_{Cad}^{\;2}\, X_{CPE} + X_{Cad}\, X_{CPE}^{\;2}}{R_{ct}^{\;2} + (X_{Cad} + X_{CPE})^2} \qquad (10),$$

$$|Z_{sim}| = \sqrt{Z_{real}^{\;2} + Z_{img}^{\;2}} \qquad (11),$$

$$\theta_{sim} = -\tan^{-1}\left(\frac{Z_{img}}{Z_{real}}\right) \qquad (12)$$

The surface area of the microelectrodes with ZnO NWs was calculated using the equation below.

$$S = \left(\frac{1}{4}\, D^2 + D\, L\right) \sigma_{NWs}\, \pi^2\, R^2 \qquad (13),$$

where $D$ and $L$ were the diameter and length of the nanowires, respectively, $\sigma_{NWs}$ was the density of the nanowires, and $R$ was the radius of the hole (25 µm) in the SU8 passivation layer concentric with the ZnO NW microelectrode. The diameter, length and density of the ZnO NWs were measured from SEM images using the ImageJ program.
