# Supplementary material for: Synthesis of encapsulated ZnO nanowires provide low impedance alternatives for microelectrodes
Source: PLoS One. 2022 Jun 16;17(6):e0270164. doi: 10.1371/journal.pone.0270164 (PMC9202946; doi:10.1371/journal.pone.0270164)
Supplement: Attachment — Submitted filename: Response to Reviewers.docx [file pone.0270164.s007.docx]

Response to the PLOS One Reviewers’ comments

**Response:** We would like to thank the journal and the reviewers for their time to review our manuscript and their positive feedback.

# Journal Requirements:

When submitting your revision, we need you to address these additional requirements.

1. Please ensure that your manuscript meets PLOS ONE's style requirements, including those for file naming. The PLOS ONE style templates can be found at
   <https://journals.plos.org/plosone/s/file?id=wjVg/PLOSOne_formatting_sample_main_body.pdf> and 
   <https://journals.plos.org/plosone/s/file?id=ba62/PLOSOne_formatting_sample_title_authors_affiliations.pdf>

**Response:** We have amended the manuscript to ensure it meets all PLOS ONE’s style requirements.

2. Thank you for stating the following in the Acknowledgments Section of your manuscript:

*"This research was supported by the Royal Society of New Zealand Marsden Fund (3709273/UOA1510). We would also greatly acknowledge the support from Victoria University of Wellington, the University of Auckland and the MacDiarmid Institute of New Zealand."*

We note that you have provided funding information that is not currently declared in your Funding Statement. However, funding information should not appear in the Acknowledgments section or other areas of your manuscript. We will only publish funding information present in the Funding Statement section of the online submission form. 
Please remove any funding-related text from the manuscript and let us know how you would like to update your Funding Statement. Currently, your Funding Statement reads as follows:

*"Mohsen Maddah: This research was supported by the Royal Society of New Zealand Marsden Fund (3709273/UOA1510)."*

Please include your amended statements within your cover letter; we will change the online submission form on your behalf.

**Response:** The current Funding Statement is correct. I have removed the funding statement from my Acknowledgements in the manuscript.

**Acknowledgements, Page 18, Paragraph 3, Lines 434–435**

*“~~This research was supported by the Royal Society of New Zealand Marsden Fund (3709273/UOA1510).~~ We would ~~also~~ greatly acknowledge the support from Victoria University of Wellington, the University of Auckland and the MacDiarmid Institute of New Zealand.”*

# Reviewer #1:

In this research article author has synthesized encapsulated ZnO nanowires which is used as a low impedance microelectrode. However, there are various shortcoming in the manuscript which are mentioned below. After major revision this manuscript is suitable for publishing.

**Response:** We would like to thank the reviewer for their time to review our manuscript and their feedback We address each of the detailed comments below.

1. XRD of synthesized ZnO nanowires is not present in the manuscript. Without XRD how author claim that the synthesized material is ZnO. XRD should be required in the revised manuscript.

**Response:** We have performed an extensive study on the ZnO nanowire synthesis and published our fabrication/characterisation of ZnO NWs synthesis (Mohsen Maddah et al 2019 Mater. Res. Express 6 015905). Rather than XRD, we have performed SEM and EDS analysis that is sufficient to claim synthesis of ZnO nanowire on microelectrodes. We have added a new figure (Fig 5) and added a new paragraph to the manuscript as shown below.

**Section 3.1 ZnO NW microelectrodes, Page 10, Lines 229–242**

*“Fig 5 shows the SEM image and energy-dispersive X-ray spectroscopy (EDS) of the fabricated ZnO NW microelectrode with the SU8 passivation layer. The EDS mapping shows silicon (Si) of the Si/SiO2 substrate and gold (Au) of the deposited Cr/Au microelectrode in Fig 5(b) and (c), respectively. Since the deposited microelectrode is 200 µm in diameter, gold is appearing everywhere in Fig 5(c). The existence of synthesised ZnO NWs is confirmed by zinc (Zn) and oxygen (O) mapping in Fig 5(d) and (e), respectively, however oxygen is also attributed to the Si/SiO2 substrate. Fig 5(f) shows carbon (C) mapping confirming the successful photolithography of the SU8 passivation layer with a 50 μm wide open window above the microelectrode.*

***Fig 5. SEM image and energy-dispersive X-ray spectroscopy (EDS) mapping of a ZnO NW microelectrode.*** *(a) SEM image of the ZnO NW microelectrode enclosed with a SU8 passivation layer. (b)–(f) EDS mapping of the ZnO NW microelectrode shown in (a). The EDS mapping shows (b) silicon (Si) of the Si/SiO2 substrate; (c) gold (Au) of the underlying Cr/Au microelectrode; (d) zinc (Zn) of ZnO nanowires; (e) oxygen (O) of ZnO nanowires and the Si/SiO2 substrate; and (f) carbon (C) of the SU8 passivation layer.”*

2. All the Figures have very low Quality. Enhancement in the quality of images is required in the manuscript.

**Response:** We have replaced all previous images with high quality images in the revised manuscript submission, as shown at the end of this document.

3. The comparison table of the synthesized materials with the previously reported literature must be required in the revised version.

**Response:** We have added a comparison table for the nanomaterial microelectrodes in the supplementary information, S1 Table as shown below, which compares our results with the existing literature. Please note that the introduced area impedance term is used for the direct comparison since the microelectrodes in the literature have different dimensions.

| Microelectrode | Nanomaterial | Nanomaterial size | Impedance (kΩ) at 1 kHz | Impedance for unit area (Ω∙cm2) | [Ref] |
| --- | --- | --- | --- | --- | --- |
| Ti/Au, 50 µm (dia) | Au nanowires | Ø: 10–1000 nm, L: 5 μm | 500 kΩ | 9.82 Ω∙cm2 | [1] |
| Cr/Ni, 30 µm (dia) | Au nanopillars | Ø: 4–200 nm, L: 4–22.5 µm | 13–360 kΩ | 0.092–2.54 Ω∙cm2 | [2] |
| Pt, 120 µm (dia) | Pt nanowires | 600× increased surface area | 2 kΩ | 0.226 Ω∙cm2 | [3] |
| Ti/Pt, 5–10 µm (sq) | Pt nanopillars | Ø: 150 nm, L: 1.5 µm | 6 MΩ | 1.5–6 Ω∙cm2 | [4] |
| Au, 50 µm (dia) | Si/SiO2 nanowires with Ti/Au tips | Ø: 150 nm, L: 3 μm | N/A | N/A | [5] |
| P-doped poly-silicon, 30 µm (sq) | Vertically aligned MW-CNTs | Ø: 30–50 nm, L: 35 µm | 3–4 kΩ | 0.027–0.036 Ω∙cm2 | [6] |
| TiN, 80 µm (dia) | MW-CNT islands | Ø: tens of nm | 1.1–10 kΩ | 0.0553–0.503 Ω∙cm2 | [7] |
| Pt, 40 µm (diam) | SW-CNTs | Ø: 10–25 nm, L: 1–1.5 µm | 20 kΩ | 0.251 Ω∙cm2 | [8,9] |
| Au,50 µm (dia) | MW-CNTs functionlised with carboxylic acid | Ø: 9.5 nm, L: 1.5 µm | 19 kΩ | 0.373 Ω∙cm2 | [10] |
| Carbon film, 30 µm (dia) | Carbon nanofiber (CNF) | Ø: 23.6 µm, L: 20.7 µm | 25 kΩ | 0.177 Ω∙cm2 | [11] |
| Au, 800 µm (sq) | Flat Au | N/A | 17.9 kΩ | 115 Ω.cm2 | [12,13] |
| Au, 800 µm (sq) | ZnO-NWs | Ø: 55 nm, L: 3.5 µm | 1.45 kΩ | 9.28 Ω.cm2 | [12,13] |
| Au, 800 µm (sq) | ZnO-NWs/ Cr/Au | Ø: 55 nm, L: 3.5 µm | 636 Ω | 4.07 Ω.cm2 | [12,13] |
| Au, 800 µm (sq) | ZnO-NWs/ Cr/Au/PEDOT | Ø: 55 nm, L: 3.5 µm | 520 Ω | 3.33 Ω.cm2 | [12,13] |
| Cr/Au, 50 µm (dia) | N/A | N/A | 835 ± 40 kΩ | 16.4 ± 0.79 Ω.cm2 | This Work |
| Cr/Au, 50 µm (dia) | ZnO-NWs | Ø: 75 ± 23 nm, L: 1.88 ± 0.178 µm | 700 ± 40 kΩ | 13.7 ± 0.79 Ω.cm2 | This Work |
| Cr/Au, 50 µm (dia) | ZnO-NWs/ Cr/Au | Ø: 75 ± 23 nm, L: 1.88 ± 0.178 µm | 680 ± 10 kΩ | 13.4 ± 0.2 Ω.cm2 | This Work |
| Cr/Au, 50 µm (dia) | ZnO-NWs/ Ti or Pt | Ø: 75 ± 23 nm, L: 1.88 ± 0.178 µm | 400 ± 25 kΩ | 7.85 ± 0.49 Ω.cm2 | This Work |
| (dia): diameter of the circular electrode, (sq): width of the square electrode, (N/A): not available/stated | | | | | |

4. How the improvement in redox reaction of the NWs affect only the Faradaic impedance.

**Response:** The Randles circuit model has commonly been used in the literature [30,87–89] to study the EIS of the microelectrodes. In this model the Faradaic impedance (Z_f_) is used as a series combination of charge transfer resistor ($R_{ct}$) and adsorption capacitor ($C_{ad}$) that is representative of all Redox reaction happening at the interface of the solution and the microelectrode. The non-Faradaic impedance attributed to the constant phase element (CPE) is responsible for the double layer capacitance behaviour at the interface of the solution and the microelectrode, and therefore does not get influenced by the redox reaction. Any contribution of the Redox reaction in EIS measurements is therefore represented by the Faradaic impedance in the Randles model. We have amended the manuscript to clarify this for the reader.

**Section 3.3 EIS Modelling, Page 14, Paragraph 2, Lines 339–351**

*“A modified Randles equivalent circuit model [30,88–90], as shown in Fig 9, was constructed to theoretically compare the EIS of the control planar and ZnO NW microelectrodes with the metal encapsulated ZnO NWs microelectrodes. The equivalent circuit comprises a series resistor (R_s_), charge transfer resistor (R_ct_), adsorption capacitor (C_ad_), and constant phase element (CPE). The series resistor (R_s_) represents all series resistance in the EIS measurement, including the resistance of the auxiliary connections, PBS solution, and underlying metallic contacts. The charge transfer resistance or the redox reaction resistance (R_ct_) represents the resistance for the charges transferring from the solution to the electrode. The adsorption capacitor (C_ad_) accounts for the adsorption of the transferred species through the double-layer interface onto the electrode surface that participates in the redox reaction. The Faradaic impedance, Z_f_, that is representative of all redox reaction at the electrode-electrolyte interface can subsequently be defined as a series combination of the charge transfer resistance and the adsorption capacitance by*

$$Z_{f} = R_{ct} + jX_{Cad}$$

(2),

*where X_Cad_ is the reactance associated with the adsorption capacitance.”*

5. How ZnO nanowires provide low impedance, authors are required to describe its mechanism.

**Response:** The main mechanism of reducing the impedance of the microelectrodes using ZnO NWs is by providing direct conduction pathways and increasing the surface area [87,90–94]. Growing ZnO NWs increases the surface area by a factor of 25× compared to a planar microelectrode. Such an increase in the surface area results in the increased redox reaction (adsorption capacitance, C_ad_) and the double-layer capacitance (CPE). Subsequently, the C_ad_ reactance measurement has decreased from 2.74 MΩ of ZnO seeds to 2.05 MΩ of ZnO NWs and the CPE reactance from 1.50 MΩ to 1.03 MΩ, respectively. The growth of ZnO NWs also increases the charge transport that provide direct conduction pathways from the solution to the underlying microelectrode. As a result, the charge transfer resistance (Rct) is reduced from 2.01 kΩ of ZnO seeds to 1.46 kΩ when ZnO NWs are grown. We have amended the entire paragraph in the manuscript to explain it more clearly.

**Section 3.3 EIS Modelling, Page 16–17, Paragraph 2, Lines 381–395**

*“The growth of ZnO NWs from the seed layer has reduced the 1 kHz impedance from 965 ± 100 kΩ of ZnO seeds to 700 ± 40 kΩ. We propose that this is primarily due to the increased surface area and, secondarily, to direct conduction pathways. The resulting ZnO NWs were measured from SEM images to have an average diameter of 75 ± 23 nm, length of 1.88 ± 0.17 µm, and density of 57 ± 6 NWs/µm^2^. The average surface area of the microelectrode was subsequently increased by a factor of 25×, from 1,960 µm^2^ of the planar microelectrode to 50,400 µm^2^ by growing ZnO NWs. The average surface area was estimated assuming ZnO NWs as cylinders, as described in S1 File. Such an increase of the microelectrode surface area has increased the adsorption capacitance (C_ad_)  that is associated with the redox reaction and the double-layer capacitance (CPE). Subsequently, the C_ad_ reactance measurement has decreased from 2.74 MΩ of the microelectrode with ZnO seed layer to 2.05 MΩ of ZnO NWs and the CPE reactance from 1.50 MΩ to 1.03 MΩ, respectively. Furthermore, the growth of ZnO NWs is thought to increase the electron transport by providing the direct conduction pathways from the nanowire surface to the underlying microelectrodes [87,90–94]. As a result, the charge transfer resistance (R_ct_) has been reduced from 2.01 kΩ of ZnO seeds to 1.46 kΩ when ZnO NWs were grown on microelectrodes.”*

6. Authors had tried to explain the results of Figure 6, but the explanation is not sufficient related with these results.

**Response:** We have amended the manuscript to better explain Fig 6 (New Fig 8), as shown below.

**Section 3.2 Metal encapsulated ZnO NW microelectrodes, Paragraph 2, Page 13, Lines 312–325**

*“Fig 8 shows the EIS Bode plots of microelectrodes with ZnO NWs that were encapsulated with layers of different metals: Cr/Au, Ti, and Pt. Fig 8(a) and (b) show that the impedance magnitudes of the Ti and Pt encapsulated ZnO NW microelectrodes were lower, particularly at low frequencies of 40 Hz–2 kHz, compared to the control planar microelectrodes and the ZnO NW microelectrodes with no metal coating. Applying ZnO NWs encapsulated with a 10 nm thin layer of Ti or Pt onto the microelectrodes improved the impedance by a factor of 2×, from 835 ± 40 kΩ of planar microelectrodes to 400 ± 25 kΩ at 1 kHz of frequency. While Ti and Pt coating resulted in improved impedance, the impedance of Cr/Au encapsulated ZnO NW microelectrodes, with an average impedance magnitude of 680 ± 10 kΩ and phase of −84° ± 5°, was very similar to the pristine ZnO NW microelectrodes, with an average impedance magnitude of 700 ± 40 kΩ and phase of −85° ± 2° at 1 kHz. Furthermore, the impedance phase of the Ti and Pt encapsulated ZnO NW microelectrodes were determined to be higher for the frequencies below 100 kHz compared to the other microelectrodes, as shown in Fig 8(c). The impedance phase of Ti and Pt encapsulated ZnO NW microelectrodes were measured to be −77° ± 2° and −60° ± 4° at 1 kHz, respectively, as shown in Fig 8(d).”*

7. In the section of “Modified Randles equivalent circuit model of microelectrodes for electrochemical impedance modelling” author have not given any reference. And the explanation of these equivalent circuit needs some support from the previously reported work.

**Response:** The relevant references in introducing the modified Randles model are included now, as below.

**Section 3.3 EIS Modelling, Page 14, Paragraph 2, Lines 339–351**

*“A modified Randles equivalent circuit model [30,88–90], as shown in Fig 9, was constructed to theoretically compare the EIS of the control planar and ZnO NW microelectrodes with the metal encapsulated ZnO NWs microelectrodes. The equivalent circuit comprises a series resistor (R_s_), charge transfer resistor (R_ct_), adsorption capacitor (C_ad_), and constant phase element (CPE). The series resistor (R_s_) represents all series resistance in the EIS measurement, including the resistance of the auxiliary connections, PBS solution, and underlying metallic contacts. The charge transfer resistance or the redox reaction resistance (R_ct_) represents the resistance for the charges transferring from the solution to the electrode. The adsorption capacitor (C_ad_) accounts for the adsorption of the transferred species through the double-layer interface onto the electrode surface that participates in the redox reaction. The Faradaic impedance, Z_f_, that is representative of all redox reaction at the electrode-electrolyte interface can subsequently be defined as a series combination of the charge transfer resistance and the adsorption capacitance by*

$$Z_{f} = R_{ct} + jX_{Cad}$$

(2),

*where X_Cad_ is the reactance associated with the adsorption capacitance.”*

8. Add some latest reference in the manuscript like ACS Applied Electronic Materials 2, 3522−3529: ECS Journal of Solid State Science and Technology 10, 023002: IEEE Journal of Photovoltaics 10 (6), 1744-1749: Journal of Alloys and Compounds 814, 152292: Optical Materials 79, 115-119

**Response:** We really appreciate the author for suggesting latest references and we have included them all in our manuscript, [51, 55, 56, 63, 64].

**Section 1 Introduction, Page 3, Paragraph 3, Lines: 69–72**

*“A more recent introduction to nanowire technology has been the introduction of vertical zinc oxide nanowires (ZnO NWs), an n-type semiconductor, commonly used in light-emitting diodes (LEDs) [50,51], solar cells [52–56], biosensors [57–59], piezoelectric devices [60–62] and sensing applications [63,64] due to their excellent electronic and optoelectronic properties.”*

# Reviewer #2:

Authors carried out their work on -Synthesis of encapsulated ZnO nanowires provide low impedance alternatives for microelectrodes. The manuscript is technically good but there are certain queries that need to be addressed as follows:

**Response:** We would like to thank the reviewer for their time to review our manuscript and their feedback. We address each of the detailed comments below.

1. Authors should give a detailed description of the fabrication step of the microelectrode as the complete manuscript revolves around it.

**Response:** We have added a new Fig 1 and have amended the manuscript accordingly using both Fig 1 and Fig 2 to describe the fabrication steps more clearly, as below:

**Section 2.1 Fabrication, Page 4-7, Lines: 96–173**

*“We fabricated microelectrodes in three different configurations: as a planar Cr/Au microelectrode, as a pristine unfunctionalised vertical ZnO NW microelectrode and as metal encapsulated ZnO NW microelectrodes (Cr/Au-ZnO NW, Ti-ZnO NW and Pt-ZnO NW). The planar microelectrode and pristine ZnO NW microelectrodes were used as controls to investigate the characteristics of the Cr/Au-ZnO NW, Ti-ZnO NW and Pt-ZnO NW microelectrodes. All of the microelectrodes were fabricated on 15 mm × 15 mm silicon substrates with a 100 nm oxide layer (Si/SiO_2_) (University Wafer) in the same configuration, as shown in Fig 1(a). The layout consisted of 32 central working microelectrodes in a 6 × 6 matrix within an area of 4 × 4 mm^2^, which were connected to the outer electrode contact pads. The microelectrodes were 200 µm in diameters with spacings of 700 µm in between.*

***Fig 1. (a) Microelectrode configuration. (b) SEM image of fabricated planar microelectrode with a SU8 passivation layer.*** *Inset in (b) shows an SEM image of the planar microelectrode overview without the SU8 passivation layer. The SEM images were taken at 70° tilted-view.*

***2.1.1 Planar microelectrode fabrication***

*The initial fabrication stages of all microelectrodes involved the production of planar Cr/Au microelectrodes without a SU8 passivation layer, as shown in Fig 1(b). Fig 2 shows the fabrication process of all microelectrodes. The substrates were initially cleaned thoroughly by sonication in acetone (one minute), sonication in isopropanol (IPA) (one minute), rinsing in IPA and then drying in a stream of clean nitrogen (N_2_). Photolithography was then carried out by coating the substrate with a thin layer of AZ1518 photoresist (MicroChemicals) via spin-coating at 4000 rpm for one minute followed by soft-baking at 95°C for two minutes on a hot-plate. A Karl Suss MJB3 mask aligner is then used to expose defined areas of the photoresist to the UV (250 mJ/cm^2^). The photoresist development was carried out by agitating in a 1:4 dilution of AZ351B developer (MicroChemicals) to deionised (DI) water for 15–20 seconds followed by 10 seconds of rinsing in DI-water and drying with N_2_ to define microelectrode patterns, as shown in Fig 2(b). The microelectrodes were deposited via thermal evaporation of 5 nm chrome followed by 50 nm of gold (Cr/Au) onto the Si/SiO_2_ substrates, using an Angstrom Engineering Nexdep Evaporator. A lift-off process was then applied using acetone and IPA to remove photoresist with the excessive Cr/Au, leaving a pattern of Cr/Au electrodes with central working microelectrodes (200 µm in diameters) on the substrates to create planar microelectrodes, as shown in Fig 2(c).*

***Fig 2. The fabrication process of the planar microelectrode, pristine ZnO NW microelectrode and metal encapsulated ZnO NW microelectrodes.*** *(a) Si/SiO_2_ substrate preparation; (b) photolithography; (c) deposition of Cr/Au layer by thermal evaporation followed by lift-off, resulting in a planar microelectrode; (d) photolithography; (e) Sputter deposition of a ZnO seed layer followed by lift-off; (f) photolithography; (g) hydrothermal growth of ZnO nanowires; (h) lift-off to remove ZnO nanowire debris, resulting in a ZnO NW microelectrode; (i) photolithography; (j) deposition of a metallic encapsulation layer by thermal or e-beam evaporation followed by lift-off, resulting in a metal encapsulated ZnO NW microelectrode. The fabrication of all microelectrodes was completed by SU8 photolithography, as shown from the top-view in (k), which deposited a SU8 passivation layer to cover the entire substrate surface except the regions above the working microelectrodes and outer electrodes for electrical measurements.*

***2.1.2 ZnO NW microelectrode fabrication***

*ZnO NW microelectrodes were fabricated directly onto the planar Cr/Au microelectrodes, as shown schematically in Fig 2(c–h). Photolithography was carried out, again using the AZ1518 photoresist and the Karl Suss MJB3 mask aligner, to define a circular gap of 100 µm diameter above the Cr/Au microelectrodes. A 100 nm thick ZnO seed layer was deposited by sputter deposition, using an HHV Auto 500 RF sputter coater, followed by a lift-off process to leave ZnO seeds on the central working microelectrodes, as shown in Fig 2(e). A third photolithography step was applied using a T-profile AZ5214E (MicroChemicals) recipe by spin-coating at 4000 rpm for one minute, soft-baking at 95° for two minutes, UV flash exposure (2.8 mJ/cm^2^), baking at 110° for two minutes, UV mask exposure (224 mJ/cm^2^), development in 1:4 dilution of AZ351B developer to DI-water for 15–20 seconds followed by 10 seconds rinsing in DI-water and drying with N_2_ to define a pattern of open areas with diameters of 200 µm concentric with the pre-deposited ZnO seeds, as shown in Fig 2(f).*

*Hydrothermal synthesis of ZnO NWs, at 95°C, was then carried out to grow ZnO NWs from the pre-deposited ZnO seed layers. The growth solution was prepared by mixing 25 mM equimolar concentration of zinc nitrate hexahydrate (98%, Sigma Aldrich) and hexamethylenetetramine (HMT 99%, Sigma Aldrich) precursors in 100 mL of DI-water. This concentration was selected to provide the optimum conditions as commonly used in the literature [67,71,80,81] for growing consistent nanowires with relatively high aspect ratios of 25 ± 8 as determined in our previous work [66]. After growing ZnO NWs for 4 h, the samples were cleaned in DI-water and the photoresist was removed by immersion in n-methyl-2-pyrrolidinone (NMP) for 2 h. This was then followed by two minutes of sonication in NMP, two minutes of sonication in IPA, rinsing in IPA and drying with N_2_. These steps together then resulted in ZnO NW microelectrodes as indicated in Fig 2(h).*

***2.1.3 Metal encapsulated ZnO NW microelectrode fabrication***

*Metal encapsulated ZnO NW microelectrodes were fabricated by an additional fabrication step on the ZnO NW microelectrodes, as depicted schematically in Fig 2(h–j). Photolithography was again carried out using the AZ1518 photoresist to define open areas with diameters of 200 µm aligned above the ZnO NWs on the central working microelectrodes, as shown in Fig 2(i). Encapsulation layers of either Cr/Au (2/20 nm), Ti (10 nm) or Pt (10 nm) were deposited by thermal or e-beam evaporation, using the Angstrom Engineering Nexdep Evaporator. A lift-off process was then applied to remove the excessive deposited materials, resulting in microelectrodes with metal encapsulated ZnO NWs, as shown in Fig 2(j).*

*The fabrication of all microelectrodes was completed by deposition of 8 µm thick SU8-2150 (MicroChemicals) as a passivation layer on microelectrodes through a photolithography process, as shown in Fig 2(k). The SU8 layer covered the entire substrate except for the regions above the working microelectrodes (with diameters of 50 µm) and the outer electrodes to allow for electrical measurements. Furthermore, the 8 µm thick SU8 passivation layer assured that only vertically grown ZnO NWs at the centre of the working microelectrodes were exposed for the electrochemical measurements. The samples were finally hard-baked at 200°C for 15 minutes to improve the chemical and physical stability of the devices.”*

2. Experiment conditions should be optimized, including the pH values and concentration of precursors.

**Response:** We have previously done a comprehensive study on the synthesis of ZnO nanowires with various parameters and it has been published (Mohsen Maddah et al 2019 Mater. Res. Express 6 015905). We have amended the manuscript and cited our paper in the fabrication process.

**Section 2.1.2 ZnO NW microelectrode fabrication, Page 6–7, Paragraph 3, Lines: 147–152**

*“Hydrothermal synthesis of ZnO NWs, at 95°C, was then carried out to grow ZnO NWs from the pre-deposited ZnO seed layers. The growth solution was prepared by mixing 25 mM equimolar concentration of zinc nitrate hexahydrate (98%, Sigma Aldrich) and hexamethylenetetramine (HMT 99%, Sigma Aldrich) precursors in 100 mL of DI-water. This concentration was selected to provide the optimum conditions as commonly used in the literature [67,71,80,81] for growing consistent nanowires with relatively high aspect ratios of 25 ± 8 as determined in our previous work [66].”*

3. Figure 1 is very poorly described. This figure should be better described.

**Response:** We have added a new Fig 1 and have amended the manuscript accordingly using both Fig 1 and Fig 2 to describe the fabrication steps more clearly, as included in the response to the first comment.

4. SEM analysis needs more elaboration. Why were some nanowires protruding from the outer edges of the seed layer?

**Response:** The growth of ZnO nanowires is thoroughly studied in our previously published paper (Mohsen Maddah et al 2019 Mater. Res. Express 6 015905).), including why ZnO nanowires are protruding from the outer edges of the seed layer. In brief, the outer edges of the ZnO seed layer are exposed to the growth solution as well as the top surface. Hence, we are observing ZnO nanowires growing in brush-head configuration at the edge of the seed layer. Nevertheless, applying the SU8 passivation layer ensures that only the vertically grown ZnO nanowires at the centre of the microelectrodes are exposed for the EIS measurements. We have amended the manuscript to clarify that only vertically grown ZnO NWs are exposed through the SU8 passivation windows.

**Section 2.1.3 Metal encapsulated ZnO NW microelectrode fabrication, Page 7, Paragraph 3, Lines: 166–173**

*“The fabrication of all microelectrodes was completed by deposition of 8 µm thick SU8-2150 (MicroChemicals) as a passivation layer on microelectrodes through a photolithography process, as shown in Fig 2(k). The SU8 layer covered the entire substrate except for the regions above the working microelectrodes (with diameters of 50 µm) and the outer electrodes to allow for electrical measurements. Furthermore, the 8 µm thick SU8 passivation layer assured that only vertically grown ZnO NWs at the centre of the working microelectrodes were exposed for the electrochemical measurements. The samples were finally hard-baked at 200°C for 15 minutes to improve the chemical and physical stability of the devices.”*

**Section 3.1 ZnO NW microelectrode, Page 9, Paragraph 2, Lines: 217–225**

*“Fig 4(a) shows SEM images of ZnO NWs that were grown from a seed layer on the microelectrode. The SEM shows that the ZnO NWs have been grown uniformly with an average diameter of 75 ± 23 nm, length of 1.88 ± 0.17 µm, and density of 57 ± 6 NWs/µm2 within the 100 μm region. While the majority of ZnO NWs were vertically aligned, some NWs were protruding from the outer edges of the seed layer exposed to the growth solution, as shown in Fig 4(a) inset. Fig 4(b) shows the ZnO NW microelectrode after photolithography of the SU8 passivation layer. The SU8 passivation layer covered the entire substrate surface with an open window of 50 µm in diameter above the microelectrode to ensure that only the vertical ZnO NWs within the 50 µm wide area were exposed to the solution for EIS.”*

5. Why the TEM and SEAD analyses were not done? Why the point-EDS analyses were not done?
6. XRD should also be included in the manuscript.

**Response:** We have performed an extensive study on the ZnO nanowire synthesis and published our fabrication/characterisation of ZnO NWs synthesis (Mohsen Maddah et al 2019 Mater. Res. Express 6 015905). Rather than XRD, we have performed SEM and EDS analysis that is sufficient to claim synthesis of ZnO nanowire on microelectrodes. We have added a new figure (Fig 5) and added a new paragraph to the manuscript as shown below.

**Section 3.1 ZnO NW microelectrodes, Page 10, Lines 229–242**

*“Fig 5 shows the SEM image and energy-dispersive X-ray spectroscopy (EDS) of the fabricated ZnO NW microelectrode with the SU8 passivation layer. The EDS mapping shows silicon (Si) of the Si/SiO2 substrate and gold (Au) of the deposited Cr/Au microelectrode in Fig 5(b) and (c), respectively. Since the deposited microelectrode is 200 µm in diameter, gold is appearing everywhere in Fig 5(c). The existence of synthesised ZnO NWs is confirmed by zinc (Zn) and oxygen (O) mapping in Fig 5(d) and (e), respectively, however oxygen is also attributed to the Si/SiO2 substrate. Fig 5(f) shows carbon (C) mapping confirming the successful photolithography of the SU8 passivation layer with a 50 μm wide open window above the microelectrode.*

***Fig 5. SEM image and energy-dispersive X-ray spectroscopy (EDS) mapping of a ZnO NW microelectrode.*** *(a) SEM image of the ZnO NW microelectrode enclosed with a SU8 passivation layer. (b)–(f) EDS mapping of the ZnO NW microelectrode shown in (a). The EDS mapping shows (b) silicon (Si) of the Si/SiO2 substrate; (c) gold (Au) of the underlying Cr/Au microelectrode; (d) zinc (Zn) of ZnO nanowires; (e) oxygen (O) of ZnO nanowires and the Si/SiO2 substrate; and (f) carbon (C) of the SU8 passivation layer.”*

7. Authors should add high quality figures in the manuscript.

**Response:** We have replaced all previous images with high quality images in the revised manuscript submission, as shown at the end of this document.

8. Authors should explain the reasons that lead to lowest electrochemical impedance on encapsulation of ZnO NW microelectrodes with thin layer of Ti or Pt?

**Response:** The dominant factor of the improved electrochemical impedance for the Ti and Pt encapsulated ZnO NWs is thought to be the non-faradaic impedance, due to the increased surface area of the NWs. The SEM and TEM images in S3 Fig show that the Ti and Pt encapsulation has appeared as a rough surface on the NWs that increase the surface area of the nanowires exposed to the electrolyte. As a result of the increased surface area, the constant phase element (non-Faradaic double layer capacitance, CPE) has increased that lowered the non-Faradaic impedance and subsequently lowered the overall electrochemical impedance of the encapsulated NW microelectrodes. This has been explained as shown below:

**Section 3.3 EIS Modelling, Page 17, Paragraph 3, Lines: 403–415**

*“The non-Faradaic impedance of the metal encapsulated ZnO NWs was determined to be the dominant factor that improved the EIS of the microelectrodes. The application of Ti and Pt encapsulation on the ZnO NWs reduced the 1 kHz non-Faradaic impedance from 1.03 MΩ of the pristine ZnO NWs to 432 kΩ and 474 kΩ, respectively, which improved their overall electrochemical impedance. The SEM and TEM micrographs in S3 Fig(b, c) show that applying a 10 nm thin encapsulation layer of Pt and Ti created a non-uniform layer on the NW surfaces that further increased the overall surface area and subsequently reduced the non-Faradaic impedance. The Cr/Au encapsulation of ZnO NWs also reduced the non-Faradaic impedance to 871 kΩ, but with less impact compared to the Ti and Pt encapsulation. As a thick layer was deposited for the Cr/Au encapsulation (2/20 nm), the encapsulation layer could cover the NWs more uniformly (lower porosity), as shown by SEM in S3 Fig(a). Applying the thick encapsulation layer of Cr/Au on the NWs could also fuse the adjacent NWs, particularly at the top regions that reduced the overall surface area of the Cr/Au encapsulated NWs compared to the Ti and Pt encapsulated NWs.”*

9. Authors should also encapsulate ZnO NW microelectrode with thin layer of Au, as their control study.

**Response:** We thank the reviewer for the suggestion. We have applied Cr/Au encapsulation mainly for comparison with the previous literature [71,79]. While it would be interesting to see how thin Au encapsulation of ZnO NW will influence the EIS of the microelectrodes, we are currently unable to do this work, but we will consider it for the next PhD candidate working in this area.

10. Some related papers based on nanowires and electrochemical sensors should be cited to give a broader view of the corresponding research field such as <https://doi.org/10.1016/j.microc.2020.104858>,

<https://doi.org/10.1149/1945-7111/abdee8>, <https://doi.org/10.1016/j.jmrt.2020.10.024>,

<https://doi.org/10.1007/s00339-018-1968-8>.

**Response:** We really appreciate the author for suggesting the latest references and we have included them in our manuscript, [40–43].

**Section 1 Introduction, Page 3, Paragraph 3, Lines: 58–62**

*“This has led to the exploitation of 3D nanomaterials to improve the impedance of microelectrodes. Gold nanostructures [13–17], carbon nanotubes (CNTs) [21,33,34], vertically aligned silicon nanowires (Si NWs) [35–37], vertically aligned platinum nanowires (Pt NWs) [38,39] and tin oxide nanowires (SnO_2_ NWs) [40–43] are the most common nanomaterials that have been used to increase surface area.”*

# Reviewer #3:

Synthesis of encapsulated ZnO nanowires provide low impedance alternatives for microelectrodes

Comments:

The author presented the synthesis of Ti and Pt encapsulated ZnO nanowires (Ti-ZnO NWs and Pt-ZnO NWs). And their application as Microelectrodes has been presented.

The manuscript is fascinating and well prepared.

My comments are not critical but could help to improve the manuscript.

**Response:** We would like to thank the reviewer for their time to review our manuscript and their positive feedback We address each of the detailed comments below.

1. In abstract, author mentioned the “seminal work of Ryu et al. by demonstrating h….” (Page 7, line 20). I think it is good to not to mention the reference. It could cover in the last of introduction part.

**Response:** We thank the reviewer for addressing this. We have removed the reference in the abstract as suggested.

**Abstract, Page 1, Paragraph 1, Lines: 25–27**

*“In this article, we ~~expand the seminal work of Ryu et al. by demonstrating~~ demonstrate how to synthesise metal encapsulated ZnO nanowires (Cr/Au-ZnO NWs, Ti-ZnO NWs and Pt-ZnO NWs) to reduce the impedance of the microelectrodes.”*

2. It is a bit confusing about the control sample. Why there are three control samples, planar Cr/Au, pristine ZnO NWs and Cr/Au-ZnO NWs)?

**Response:** We have amended the entire manuscript to only state planar Cr/Au and pristine ZnO NW microelectrodes as control samples in which the Cr/Au-ZnO NWs, Ti-ZnO NW and Pt-metal encapsulated ZnO NW microelectrodes (Cr/Au, Ti and Pt) are compared to.

3. The synthesis part is a bit confusing. It could be good to prepare a separate section in experimental details part to cover the synthesis part.

**Response:** We have added a new Fig 1 and have amended the manuscript accordingly using both Fig 1 and Fig 2 to describe the fabrication steps more clearly, as below:

**Section 2.1 Fabrication, Page 4-7, Lines: 96–173**

*“We fabricated microelectrodes in three different configurations: as a planar Cr/Au microelectrode, as a pristine unfunctionalised vertical ZnO NW microelectrode and as metal encapsulated ZnO NW microelectrodes (Cr/Au-ZnO NW, Ti-ZnO NW and Pt-ZnO NW). The planar microelectrode and pristine ZnO NW microelectrodes were used as controls to investigate the characteristics of the Cr/Au-ZnO NW, Ti-ZnO NW and Pt-ZnO NW microelectrodes. All of the microelectrodes were fabricated on 15 mm × 15 mm silicon substrates with a 100 nm oxide layer (Si/SiO_2_) (University Wafer) in the same configuration, as shown in Fig 1(a). The layout consisted of 32 central working microelectrodes in a 6 × 6 matrix within an area of 4 × 4 mm^2^, which were connected to the outer electrode contact pads. The microelectrodes were 200 µm in diameters with spacings of 700 µm in between.*

***Fig 1. (a) Microelectrode configuration. (b) SEM image of fabricated planar microelectrode with a SU8 passivation layer.*** *Inset in (b) shows an SEM image of the planar microelectrode overview without the SU8 passivation layer. The SEM images were taken at 70° tilted-view.*

***2.1.1 Planar microelectrode fabrication***

*The initial fabrication stages of all microelectrodes involved the production of planar Cr/Au microelectrodes without a SU8 passivation layer, as shown in Fig 1(b). Fig 2 shows the fabrication process of all microelectrodes. The substrates were initially cleaned thoroughly by sonication in acetone (one minute), sonication in isopropanol (IPA) (one minute), rinsing in IPA and then drying in a stream of clean nitrogen (N_2_). Photolithography was then carried out by coating the substrate with a thin layer of AZ1518 photoresist (MicroChemicals) via spin-coating at 4000 rpm for one minute followed by soft-baking at 95°C for two minutes on a hot-plate. A Karl Suss MJB3 mask aligner is then used to expose defined areas of the photoresist to the UV (250 mJ/cm^2^). The photoresist development was carried out by agitating in a 1:4 dilution of AZ351B developer (MicroChemicals) to deionised (DI) water for 15–20 seconds followed by 10 seconds of rinsing in DI-water and drying with N_2_ to define microelectrode patterns, as shown in Fig 2(b). The microelectrodes were deposited via thermal evaporation of 5 nm chrome followed by 50 nm of gold (Cr/Au) onto the Si/SiO_2_ substrates, using an Angstrom Engineering Nexdep Evaporator. A lift-off process was then applied using acetone and IPA to remove photoresist with the excessive Cr/Au, leaving a pattern of Cr/Au electrodes with central working microelectrodes (200 µm in diameters) on the substrates to create planar microelectrodes, as shown in Fig 2(c).*

***Fig 2. The fabrication process of the planar microelectrode, pristine ZnO NW microelectrode and metal encapsulated ZnO NW microelectrodes.*** *(a) Si/SiO_2_ substrate preparation; (b) photolithography; (c) deposition of Cr/Au layer by thermal evaporation followed by lift-off, resulting in a planar microelectrode; (d) photolithography; (e) Sputter deposition of a ZnO seed layer followed by lift-off; (f) photolithography; (g) hydrothermal growth of ZnO nanowires; (h) lift-off to remove ZnO nanowire debris, resulting in a ZnO NW microelectrode; (i) photolithography; (j) deposition of a metallic encapsulation layer by thermal or e-beam evaporation followed by lift-off, resulting in a metal encapsulated ZnO NW microelectrode. The fabrication of all microelectrodes was completed by SU8 photolithography, as shown from the top-view in (k), which deposited a SU8 passivation layer to cover the entire substrate surface except the regions above the working microelectrodes and outer electrodes for electrical measurements.*

***2.1.2 ZnO NW microelectrode fabrication***

*ZnO NW microelectrodes were fabricated directly onto the planar Cr/Au microelectrodes, as shown schematically in Fig 2(c–h). Photolithography was carried out, again using the AZ1518 photoresist and the Karl Suss MJB3 mask aligner, to define a circular gap of 100 µm diameter above the Cr/Au microelectrodes. A 100 nm thick ZnO seed layer was deposited by sputter deposition, using an HHV Auto 500 RF sputter coater, followed by a lift-off process to leave ZnO seeds on the central working microelectrodes, as shown in Fig 2(e). A third photolithography step was applied using a T-profile AZ5214E (MicroChemicals) recipe by spin-coating at 4000 rpm for one minute, soft-baking at 95° for two minutes, UV flash exposure (2.8 mJ/cm^2^), baking at 110° for two minutes, UV mask exposure (224 mJ/cm^2^), development in 1:4 dilution of AZ351B developer to DI-water for 15–20 seconds followed by 10 seconds rinsing in DI-water and drying with N_2_ to define a pattern of open areas with diameters of 200 µm concentric with the pre-deposited ZnO seeds, as shown in Fig 2(f).*

*Hydrothermal synthesis of ZnO NWs, at 95°C, was then carried out to grow ZnO NWs from the pre-deposited ZnO seed layers. The growth solution was prepared by mixing 25 mM equimolar concentration of zinc nitrate hexahydrate (98%, Sigma Aldrich) and hexamethylenetetramine (HMT 99%, Sigma Aldrich) precursors in 100 mL of DI-water. This concentration was selected to provide the optimum conditions as commonly used in the literature [67,71,80,81] for growing consistent nanowires with relatively high aspect ratios of 25 ± 8 as determined in our previous work [66]. After growing ZnO NWs for 4 h, the samples were cleaned in DI-water and the photoresist was removed by immersion in n-methyl-2-pyrrolidinone (NMP) for 2 h. This was then followed by two minutes of sonication in NMP, two minutes of sonication in IPA, rinsing in IPA and drying with N_2_. These steps together then resulted in ZnO NW microelectrodes as indicated in Fig 2(h).*

***2.1.3 Metal encapsulated ZnO NW microelectrode fabrication***

*Metal encapsulated ZnO NW microelectrodes were fabricated by an additional fabrication step on the ZnO NW microelectrodes, as depicted schematically in Fig 2(h–j). Photolithography was again carried out using the AZ1518 photoresist to define open areas with diameters of 200 µm aligned above the ZnO NWs on the central working microelectrodes, as shown in Fig 2(i). Encapsulation layers of either Cr/Au (2/20 nm), Ti (10 nm) or Pt (10 nm) were deposited by thermal or e-beam evaporation, using the Angstrom Engineering Nexdep Evaporator. A lift-off process was then applied to remove the excessive deposited materials, resulting in microelectrodes with metal encapsulated ZnO NWs, as shown in Fig 2(j).*

*The fabrication of all microelectrodes was completed by deposition of 8 µm thick SU8-2150 (MicroChemicals) as a passivation layer on microelectrodes through a photolithography process, as shown in Fig 2(k). The SU8 layer covered the entire substrate except for the regions above the working microelectrodes (with diameters of 50 µm) and the outer electrodes to allow for electrical measurements. Furthermore, the 8 µm thick SU8 passivation layer assured that only vertically grown ZnO NWs at the centre of the working microelectrodes were exposed for the electrochemical measurements. The samples were finally hard-baked at 200°C for 15 minutes to improve the chemical and physical stability of the devices.”*

4. It is good to replace the “Our group” by mentioned the author name et al. Please check, Line number 235, Page 16.

**Response:** We thank the reviewer for addressing this. We have amended this as below:

**Section 1 Introduction, Page 4, Paragraph 1, Lines: 76–79**

*“Plank et al. [66–68] and others [69,70] have previously demonstrated how the morphology of ZnO NWs can easily be controlled by the hydrothermal growth parameters such as precursor concentration, growth time, growth temperature, ZnO seed layers, and additive auxiliary agents.”*

**Section 3.1 ZnO NW Microelectrodes, Page 12, Paragraph 1, Lines: 283–286**

*“Burke-Govey et al. [68] previously demonstrated the high conductivity of the ZnO NWs as the channel in field-effect transistors, where the conductivity of the as-grown ZnO NWs was demonstrated to be less dependent on the field compared to the similarly prepared thinner nanowires.”*

5. It is good to put the Ryu et al. and author comparative values into a Table.

**Response:** We have added a comparison table for the nanomaterial microelectrodes in the supplementary information, S1 Table as shown below, which compares our results with the existing literature. Please note that the introduced area impedance term is used for the direct comparison since the microelectrodes in the literature have different dimensions.

| Microelectrode | Nanomaterial | Nanomaterial size | Impedance (kΩ) at 1 kHz | Impedance for unit area (Ω∙cm2) | [Ref] |
| --- | --- | --- | --- | --- | --- |
| Ti/Au, 50 µm (dia) | Au nanowires | Ø: 10–1000 nm, L: 5 μm | 500 kΩ | 9.82 Ω∙cm2 | [1] |
| Cr/Ni, 30 µm (dia) | Au nanopillars | Ø: 4–200 nm, L: 4–22.5 µm | 13–360 kΩ | 0.092–2.54 Ω∙cm2 | [2] |
| Pt, 120 µm (dia) | Pt nanowires | 600× increased surface area | 2 kΩ | 0.226 Ω∙cm2 | [3] |
| Ti/Pt, 5–10 µm (sq) | Pt nanopillars | Ø: 150 nm, L: 1.5 µm | 6 MΩ | 1.5–6 Ω∙cm2 | [4] |
| Au, 50 µm (dia) | Si/SiO2 nanowires with Ti/Au tips | Ø: 150 nm, L: 3 μm | N/A | N/A | [5] |
| P-doped poly-silicon, 30 µm (sq) | Vertically aligned MW-CNTs | Ø: 30–50 nm, L: 35 µm | 3–4 kΩ | 0.027–0.036 Ω∙cm2 | [6] |
| TiN, 80 µm (dia) | MW-CNT islands | Ø: tens of nm | 1.1–10 kΩ | 0.0553–0.503 Ω∙cm2 | [7] |
| Pt, 40 µm (diam) | SW-CNTs | Ø: 10–25 nm, L: 1–1.5 µm | 20 kΩ | 0.251 Ω∙cm2 | [8,9] |
| Au,50 µm (dia) | MW-CNTs functionlised with carboxylic acid | Ø: 9.5 nm, L: 1.5 µm | 19 kΩ | 0.373 Ω∙cm2 | [10] |
| Carbon film, 30 µm (dia) | Carbon nanofiber (CNF) | Ø: 23.6 µm, L: 20.7 µm | 25 kΩ | 0.177 Ω∙cm2 | [11] |
| Au, 800 µm (sq) | Flat Au | N/A | 17.9 kΩ | 115 Ω.cm2 | [12,13] |
| Au, 800 µm (sq) | ZnO-NWs | Ø: 55 nm, L: 3.5 µm | 1.45 kΩ | 9.28 Ω.cm2 | [12,13] |
| Au, 800 µm (sq) | ZnO-NWs/ Cr/Au | Ø: 55 nm, L: 3.5 µm | 636 Ω | 4.07 Ω.cm2 | [12,13] |
| Au, 800 µm (sq) | ZnO-NWs/ Cr/Au/PEDOT | Ø: 55 nm, L: 3.5 µm | 520 Ω | 3.33 Ω.cm2 | [12,13] |
| Cr/Au, 50 µm (dia) | N/A | N/A | 835 ± 40 kΩ | 16.4 ± 0.79 Ω.cm2 | This Work |
| Cr/Au, 50 µm (dia) | ZnO-NWs | Ø: 75 ± 23 nm, L: 1.88 ± 0.178 µm | 700 ± 40 kΩ | 13.7 ± 0.79 Ω.cm2 | This Work |
| Cr/Au, 50 µm (dia) | ZnO-NWs/ Cr/Au | Ø: 75 ± 23 nm, L: 1.88 ± 0.178 µm | 680 ± 10 kΩ | 13.4 ± 0.2 Ω.cm2 | This Work |
| Cr/Au, 50 µm (dia) | ZnO-NWs/ Ti or Pt | Ø: 75 ± 23 nm, L: 1.88 ± 0.178 µm | 400 ± 25 kΩ | 7.85 ± 0.49 Ω.cm2 | This Work |
| (dia): diameter of the circular electrode, (sq): width of the square electrode, (N/A): not available/stated | | | | | |

6. A few Figures are not clear. Please check their quality.

**Response:** We have replaced all previous images with high quality images in the revised manuscript submission, as shown at the end of this document.

# New Figures:

*Fig 1.*

*Fig 2.*

*Fig 3.*

*Fig 4.*

*Fig 5.*

*Fig 6.*

*Fig 7.*

*Fig 8.*

*Fig 9.*

*S1 Fig.*

*S2 Fig.*

*S3 Fig.*
